# Airway tree caliber heterogeneity and airflow obstruction among older adults

**DOI:** 10.1152/japplphysiol.00694.2022

**Published:** 2024-02-29

**Authors:** Motahareh Vameghestahbanati, Leina Kingdom, Eric A. Hoffman, Miranda Kirby, Norrina B. Allen, Elsa Angelini, Alain Bertoni, Qutayba Hamid, James C. Hogg, David R. Jacobs, Andrew Laine, Francois Maltais, Erin D. Michos, Coralynn Sack, Don Sin, Karol E. Watson, Artur Wysoczanksi, David Couper, Christopher Cooper, Meilan Han, Prescott Woodruff, Wan C. Tan, Jean Bourbeau, R. Graham Barr, Benjamin M. Smith, on behalf of investigators from the Multi-Ethnic Study of Atherosclerosis (MESA) Lung Study; the Canadian Cohort of Obstructive Lung Disease (CanCOLD); and the Subpopulations and Intermediate Outcome Measures in Chronic Obstructive Pulmonary Disease Study (SPIROMICS)

**Affiliations:** ^1^Department of Medicine, McGill University, Montreal, Quebec, Canada; ^2^Department of Radiology, University of Iowa, Iowa City, Iowa, United States; ^3^Department of Physics, Ryerson University, Toronto, Ontario, Canada; ^4^Center for Translational Metabolism and Health, Institute for Public Health and Medicine, Northwestern University, Chicago, Illinois, United States; ^5^Faculty of Medicine, Imperial College London, London, United Kingdom; ^6^Department of Medicine, Columbia University, New York, New York, United States; ^7^Department of Public Health Sciences, Wake Forest University, Winston-Salem, North Carolina, United States; ^8^Faculty of Medicine, University of Sharjah, Sharjah, United Arab Emirates; ^9^Centre for Heart Lung Innovation, University of British Columbia, Vancouver, British Columbia, Canada; ^10^School of Public Health, University of Minnesota, Minneapolis, Minnesota, United States; ^11^Faculty of Medicine , University of Laval, Laval, Quebec, Canada; ^12^Faculty of Medicine, Johns Hopkins University, Baltimore, Maryland, United States; ^13^Department of Medicine, University of Washington, Seattle, Washington, United States; ^14^Department of Medicine, University of California, Los Angeles, California, United States; ^15^Department of Biostatistics, University of North Carolina, North Carolina, United States; ^16^Division of Pulmonary and Critical Care Medicine, University of Michigan, Ann Arbor, Michigan, United States; ^17^Division of Pulmonary and Critical Care Medicine, University of California, San Francisco, California, United States

**Keywords:** airflow obstruction, airway tree caliber heterogeneity, chronic obstructive pulmonary disease, computed tomography

## Abstract

Smaller mean airway tree caliber is associated with airflow obstruction and chronic obstructive pulmonary disease (COPD). We investigated whether airway tree caliber heterogeneity was associated with airflow obstruction and COPD. Two community-based cohorts (MESA Lung, CanCOLD) and a longitudinal case-control study of COPD (SPIROMICS) performed spirometry and computed tomography measurements of airway lumen diameters at standard anatomical locations (trachea-to-subsegments) and total lung volume. Percent-predicted airway lumen diameters were calculated using sex-specific reference equations accounting for age, height, and lung volume. The association of airway tree caliber heterogeneity, quantified as the standard deviation (SD) of percent-predicted airway lumen diameters, with baseline forced expired volume in 1-second (FEV_1_), FEV_1_/forced vital capacity (FEV_1_/FVC) and COPD, as well as longitudinal spirometry, were assessed using regression models adjusted for age, sex, height, race-ethnicity, and mean airway tree caliber. Among 2,505 MESA Lung participants (means ± SD age: 69 ± 9 yr; 53% female, mean airway tree caliber: 99 ± 10% predicted, airway tree caliber heterogeneity: 14 ± 5%; median follow-up: 6.1 yr), participants in the highest quartile of airway tree caliber heterogeneity exhibited lower FEV_1_ (adjusted mean difference: −125 mL, 95%CI: −171,−79), lower FEV_1_/FVC (adjusted mean difference: −0.01, 95%CI: −0.02,−0.01), and higher odds of COPD (adjusted odds ratio: 1.42, 95%CI: 1.01–2.02) when compared with the lowest quartile, whereas longitudinal changes in FEV_1_ and FEV_1_/FVC did not differ significantly. Observations in CanCOLD and SPIROMICS were consistent. Among older adults, airway tree caliber heterogeneity was associated with airflow obstruction and COPD at baseline but was not associated with longitudinal changes in spirometry.

**NEW & NOTEWORTHY** In this study, by leveraging two community-based samples and a case-control study of heavy smokers, we show that among older adults, airway tree caliber heterogeneity quantified by CT is associated with airflow obstruction and COPD independent of age, sex, height, race-ethnicity, and dysanapsis. These observations suggest that airway tree caliber heterogeneity is a structural trait associated with low baseline lung function and normal decline trajectory that is relevant to COPD.

## Graphical abstract

**Figure:**
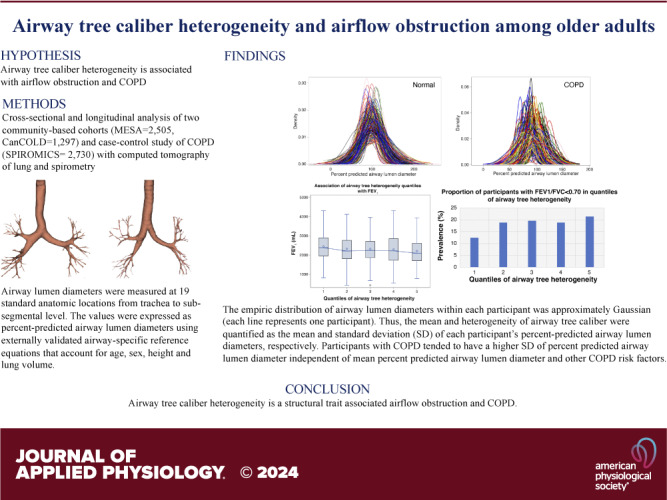

## INTRODUCTION

Chronic obstructive pulmonary disease (COPD) is a leading cause of death and disability worldwide (1). Tobacco smoking is a major COPD risk factor (2), but despite decades of declining smoking rates in many countries (3–5), the corresponding decreases in disease burden have been modest (6, 7). Although other factors have been linked to COPD (e.g., secondhand smoke, air pollutants, asthma), emerging evidence suggests that host factors, such as lung development may play a central role (2, 8, 9).

The airway tree forms early in life and variation in airway tree structure is common among adults in the general population (9). Structural properties of the airway tree influence airway resistance and flow (10–13), and smaller mean airway tree caliber quantified by computed tomography (CT) is associated with COPD independent of tobacco smoking and other risk factors, but not with accelerated lung function decline (9). This observation is consistent with the trajectory of low early-life lung function followed by normal lung function decline that accounts for up to 50% of COPD encountered later in life (14), but the relevance of other aspects of the native airway tree structure to COPD remain poorly understood.

Both computational studies of airway tree fluid dynamics and in vivo inert gas washout studies of ventilation maldistribution suggest that heterogeneity of conducting airway tree caliber contribute to obstructive lung disease pathophysiology (15–23). We note, however, that computational modeling study inputs are often informed by airway tree measurements obtained from just a handful of donor lungs with limited clinical/phenotypic characterization (24). Moreover, in vivo inert gas washout studies have made inferences about underlying mechanisms, such as conduction-dependent inhomogeneity in the central conducting airways, but rarely quantify conducting airway tree structure in vivo. Indeed, a consensus statement for inert gas washout measurement summarizing the mechanism of convection-dependent inhomogeneity in airways proximal to terminal bronchioles references a single study that quantified acinar airway dimensions rather than conducting airway dimensions from two human cadavers for which subject age, sex, and lung disease status were not reported (25, 26). A small but growing number of hyperpolarized gas imaging studies have overcome some of these limitations and demonstrated that regional gas distribution deficits tend to correlate with corresponding conducting airway lumen caliber (27–31) but the empiric distribution of conducting airway tree caliber heterogeneity in the general population and its potential clinical relevance to spirometry-assessed airflow obstruction independent of mean airway tree caliber remains uncertain.

This study sought to characterize the extent of airway tree caliber heterogeneity among nonsmoking adults free of standard COPD risk factors using CT. Next, we tested the hypothesis that airway tree caliber heterogeneity would be associated with baseline airflow obstruction and COPD independent of mean airway tree caliber but would not be associated with prospective lung function decline.

## METHODS

### Study Participants

Data from two community-based cohorts [the Multi-Ethnic Study of Atherosclerosis (MESA) Lung Study and the Canadian Cohort of Obstructive Lung Disease (CanCOLD)] were used to characterize airway tree caliber heterogeneity. Data from these cohorts and from a longitudinal case-control study of smokers with and without COPD [the Subpopulations and Intermediate Outcome Measures in COPD Study (SPIROMICS)] were then used to evaluate the association of airway tree caliber heterogeneity with airflow obstruction and COPD. Institutional review board approval was obtained at each study site. All participants provided written informed consent.

MESA is a prospective community-based study that recruited 6,814 non-Hispanic whites, African Americans, Hispanics, and Chinese Americans 45–84 yr of age in 2000–2002 (*exam 1*) from the general population in six US communities (32). MESA excluded individuals if they had a clinical diagnosis of cardiovascular disease, weight >300 pounds, or impediment to long-term follow-up at the baseline visit (2000–2002). The MESA Lung Study enrolled participants sampled from MESA who consented to genetic analyses and completed an examination in 2004–2006 (*exam 3/4*) (33) and all participants in the MESA Air Study, which enrolled additional participants of comparable age from the same study sites and who were free of clinical cardiovascular disease in 2005–2007 (34). The MESA Lung Study performed full-lung CT and spirometry in 2010–2012 (*exam 5*) and 2016–2018 (*exam 6*). The current study used measures obtained at MESA Visits 5 and 6 (2010–2018). Thus, participants may have developed clinical cardiovascular disease by the time of assessment.

The Canadian Chronic Obstructive Lung Disease (COLD) prevalence study used census data to recruit a random sample of noninstitutionalized adults 40 yr and older from nine Canadian communities in 2005–2009 (35). In 2010–2014, CanCOLD, a nested community-based case-control study enrolled COLD participants with COPD, in addition to representative random subsets of COLD nonsmoking participants and smoking participants without COPD matched on age and sex, performed full-lung CT and spirometry, with 18 and 36-mo follow-up assessments (2011–2017). CanCOLD excluded institutionalized individuals but did not exclude individuals based on the presence or absence of disease (35).

SPIROMICS is a longitudinal case-control study that recruited participants with and without COPD, 40–80 yr of age reporting 20+ pack-yr of smoking, in addition to nonsmoking participants, at 12 US medical centers in 2010–2015, and performed full-lung CT and spirometry with up to four follow-up assessments in 2011–2019 (36). SPIROMICS excluded individuals with chronic lung diseases other than COPD or asthma, body mass index greater than 40 kg/m^2^, or prior surgical lung resection.

### Airway Tree Caliber Heterogeneity

All participants underwent full-lung CT scan at suspended maximum inspiration according to standardized protocols (9). In all studies, total lung volume and airway lumen diameters were measured using Apollo Software (VIDA Diagnostics, Coralville, Iowa) (8, 11, 37). Briefly, airway lumen diameters at 19 standard anatomical locations (trachea, right mainstem, left mainstem, bronchus intermedius, right upper lobe, right middle lobe, right lower lobe, left upper lobe, left lower lobe, RB1, RB4, RB10, LB1, and LB10 bronchi), as well as the average airway lumen diameters of the subsegments along each of the five pre-specified paths (sRB1, sRB4, sRB10, sLB1, and sLB10) and total lung volume were measured by trained technologists unaware of other participant information. The scan rescan reproducibility of these measures was excellent (9, 38). For each of the 19 standard anatomical airways, the percent-predicted airway caliber was calculated using externally validated sex-stratified airway-specific reference equations derived from a general population sample of adults free of COPD risk factors (i.e., people who never smoked cigarettes, pipes, or cigars, who never lived or worked with someone who smoked indoors, who never worked a job that exposed them to vapor-gas dust or fumes, and who never had a diagnosis of asthma) (39). The sex-stratified airway-specific reference equations included spline terms for total lung volume, age, and body height (9). (see *Airway lumen diameter reference equations* s*ection in appendix-* https://figshare.com/s/20cb38b57ba3af1078a9). For each participant, the distribution of percent-predicted airway lumen diameters was approximately Gaussian (see results), therefore airway tree caliber heterogeneity was quantified as the standard deviation (SD) of the 19 percent-predicted airway lumen diameters. Participant mean airway tree caliber was quantified as the mean of the 19 percent-predicted airway lumen diameters.

### Airflow Obstruction and COPD

Spirometry was performed in all studies following American Thoracic Society Standards (40). COPD was defined based on a postbronchodilator forced expired volume in 1-s (FEV_1_)/forced vital capacity (FVC) <0.7 (2, 41) and lower limit of normal (42). In sensitivity analysis, COPD was defined as FEV_1_/FVC < 0.7 with respiratory symptoms (2) defined by COPD Assessment Test Score of 10 or more (range, 0–40) (43), presence of chronic bronchitis (yes/no) (44), or modified Medical Research Council dyspnea score higher than 0 (range, 0–4) (45).

### Other Variables

Age, sex, tobacco smoking status (cigarette, pipe, cigar), second-hand smoke exposure, occupational exposure status to vapor-gas dust or fumes, and physician diagnosis of asthma were self-reported. Race/ethnicity was assessed by self-report using fixed-category questionnaire items. Where questionnaire items differed by study, a harmonized variable was defined (9). Height and weight were measured using standardized protocols in all studies. Pack-years of smoking were calculated by multiplying the number of years smoked by the mean number of daily cigarettes divided by 20.

### Statistical Analysis

Participant characteristics were summarized by the study. CanCOLD participant characteristics and analyses were weighted by the inverse probability of selection from the COLD study to provide estimates representative of this population-based sample. Multiple imputation was used to account for missing postbronchodilator spirometry in MESA Lung (9). Given little missing data in the other studies, missing covariate data were assigned an indicator variable.

We first characterized airway tree caliber heterogeneity among the community-based nonsmoking participants who were free of secondhand or occupational exposures, or asthma. The distribution of the 19 percent-predicted airway lumen diameters in each participant was depicted using a kernel density function and, based on the Gaussian-like distribution (see results), participant airway tree caliber heterogeneity was quantified as the SD of percent-predicted airway lumen diameters. The Spearman correlation between airway tree caliber heterogeneity and mean airway tree caliber was computed.

Next, the associations of airway tree caliber heterogeneity with baseline FEV_1_ and FEV_1_/FVC were assessed using linear regression models. Airway tree caliber heterogeneity was analyzed by quartile (with the lowest airway tree caliber heterogeneity quartile as the reference group) and as a continuous variable. Models were adjusted for age, age^2^, sex, height, height^2^, race-ethnicity, and mean airway tree caliber (*model 1*; main model) and additionally adjusted for primary tobacco, secondhand smoke and occupational exposures, and asthma (*model 2*). The association between airway tree caliber heterogeneity and baseline COPD status was assessed using logistic regression with the same covariables described earlier.

Airway tree caliber heterogeneity associations with longitudinal change in FEV_1_ and FEV_1_/FVC were assessed using mixed model regression with random intercepts and autoregressive covariance structure and were adjusted as above with smoking status as a time-varying covariate. The product term between time and airway tree caliber heterogeneity was the variable of interest.

Sensitivity analyses included *1*) the use of restricted cubic splines to assess for nonlinear associations, *2*) analysis of the unweighted CanCOLD sample, and the unimputed MESA sample, *3*) stratification by cigarette smoking status (never/ever) in community-based samples, *4*) quantification of airway tree caliber heterogeneity using the coefficient of variation (SD of percent-predicted airway lumen diameters divided by the mean of percent-predicted airway lumen diameters), a measure that combines airway tree caliber heterogeneity and mean airway tree caliber into a single ratio measure, *5*) a race-ethnicity-stratified analysis of MESA Lung, *6*) analyses additionally adjusted for the study site, and *7*) association of airway tree caliber heterogeneity with COPD defined by spirometry and respiratory symptoms (9).

All analyses were performed using SAS 9.4 (Raleigh, NC). A two-sided *P* value < 0.05 was considered statistically significant.

## RESULTS

A flowchart of participant selection for the present analysis is depicted in Supplemental Fig. S1 (all Supplemental material is available at https://doi.org/10.6084/m9.figshare.23298158) and participant characteristics by study are summarized in Table 1.

**Table 1.** Participant characteristics by study

	MESA Lung		CanCOLD		Spiromics
	All Participants	Participants Free of Standard COPD Risk Factors^1^	All Participants	Participants Free of Standard COPD Risk Factors	Participants with 20+ pack-yr
No. of participants	2,505	289	1,297	204	2,730
Age, means ± SD yr	69 ± 9	65 ± 8	67 ± 10	67 ± 12	63 ± 9
Sex, No. (%)					
Female	1,317 (53)	169 (58)	579 (45)	92 (45)	1,253 (46)
Male	1,188 (47)	120 (42)	718 (55)	112 (55)	1,477 (54)
Height, means ± SD, cm	165 ± 10	164 ± 9	168 ± 9	167 ± 11	170 ± 10
Race-ethnicity, No. (%)					
Non-Hispanic White	972 (39)	79 (27)	1,215 (94)	183 (90)	2,018 (74)
Non-Hispanic Black	649 (26)	55 (19)	15 (1)	7 (3)	493 (18)
Hispanic	549 (22)	58 (20)	6 (0.5)	2 (0.8)	127 (5)
Non-Hispanic Chinese	335 (13)	97 (34)	44 (3)	13 (6)	26 (1)
Other	0 (0)	0 (0)	17 (1)	0 (0)	66 (2)
Smoking status, No. (%)					
Never	1,216 (49)	289 (100)	614 (47)	204 (100)	0 (0)
Former	1,073 (43)	0 (0)	511 (39)	0 (0)	1,652 (61)
Current	216 (9)	0 (0)	172 (13)	0 (0)	1,078 (39)
Pack-yr among ever-smoking participants, median (IQR)	19 (7, 37)	0 (0, 0)	19 (6, 35)	0 (0, 0)	43 (31, 60)
Pipe/cigar smoking, ever, No. (%)	186 (7)	0 (0)	159 (12)	0 (0)	230 (10)
Second-hand smoke exposure in adulthood, ever, No. (%)	1,244 (50)	0 (0)	510 (39)	0 (0)	1,136 (42)
Occupational exposure to vape, dust, gas or fume, No. (%)	987 (39)	0 (0)	117 (9)	0 (0)	1,145 (42)
Asthma diagnosis ever, No. (%)	208 (8)	0 (0)	234 (18)	0 (0)	548 (20)
Baseline spirometry					
Prebronchodilator FEV_1_, L, means ± SD	2.3 ± 0.7	2.3 ± 0.7	2.5 ± 0.9	2.6 ± 0.9	2.0 ± 0.9
Prebronchodilator FEV_1_/FVC, mean ± SD	0.74 ± 0.08	0.76 ± 0.09	0.71 ± 0.08	0.74 ± 0.08	0.58 ± 0.16
COPD^2^ prevalence, No. (%)	471 (19)	27 (9)	314 (25)	33 (16)	1,816 (67)
Follow-up spirometry analysis after baseline airway tree caliber heterogeneity assessment					
No. of participants	2,505	257	1,043	172	2,144
Total follow-up interval, median (25^th^, 75^th^ percentile), y	6.1 (6.0, 6.4)	6.1 (5.8, 6.4)	3.0 (1.8, 3.3)	3.0 (1.8, 3.3)	5.0 (2.0, 6.0)
No. of follow-up spirometry assessments, median (IQR)	1 (1, 1)	1 (1,1)	2 (1, 3)	2 (1,3)	3 (2, 4)
Change in FEV_1_/FVC, means ± SD/yr	−0.00 ± 0.01	−0.00 ± 0.01	−0.00 ± 0.02	−0.00 ± 0.02	−0.01 ± 0.03
Change in FEV_1_, means ± SD, mL/yr	−33 ± 31	−31 ± 35	−36 ± 75	−30 ± 87	−40 ± 137
Percent-predicted airway tree caliber,^3^ means ± SD	99 ± 10	99 ± 9	96 ± 9	98 ± 12	98 ± 11

Airway tree caliber heterogeneity was quantified as the standard deviation (SD) of percent-predicted airway diameters measured at 19 standard anatomic locations. COPD, chronic obstructive lung disease; CanCOLD, Canadian cohort of obstructive lung disease; FEV_1_, forced expired volume in 1 second; FVC, forced vital capacity; MESA, Multi-Ethnic study of atherosclerosis; SPIROMICS, Subpopulations and Intermediate Outcome Measures in COPD Study.

^1^Standard COPD risk factors included tobacco smoking (cigarettes, pipes, or cigars), occupational exposure to vapor-gas dust or fumes, or asthma.

^2^COPD is defined by postbronchodilator FEV_1_/FVC < 0.7.

^3^The percent-predicted airway tree caliber for each participant was quantified as the geometric mean of percent-predicted airway lumen diameters measured at 19 standard anatomical locations defined by externally validated sex-stratified airway-specific lumen diameter reference equations with terms for total lung volume, age, and height.

Among the 2,505 included MESA Lung participants, the means ± SD age was 69 ± 9 yr, 53% were female, and race-ethnic proportions were 39% non-Hispanic white, 26% non-Hispanic black, 22% Hispanic, and 13% non-Hispanic Chinese. The mean FEV_1_/FVC was 0.74 ± 0.08 and mean airway tree caliber was 99 ± 10% predicted. Over a median of 6.1-yr follow-up interval, the change in FEV_1_ and FEV_1_/FVC was −33 ± 31 mL/yr and −0.002 ± 0.008 per yr, respectively. Compared with included participants, excluded MESA participants differed slightly by age and smoking history (Supplemental Table S1).

Among 1,297 included CanCOLD participants, the mean age was 67 ± 10 yr, 45% were female, and 94% were non-Hispanic white. The mean FEV_1_/FVC was 0.71 ± 0.08 and mean airway tree caliber was 96 ± 9% predicted. Over a median of 3.0-yr follow-up interval, the change in FEV_1_ and FEV_1_/FVC was −36 ± 75 mL/yr and −0.003 ± 0.020 per yr, respectively. Excluded CanCOLD participants were more likely to be female and have fewer pack-years of smoking (Supplemental Table S1).

Among 2,730 included SPIROMICS participants with 20+ pack-yr of smoking history, the mean age was 63 ± 9 yr, 46% were female, 74% were non-Hispanic white, and 18% non-Hispanic black. The mean FEV_1_/FVC was 0.58 ± 0.16 and mean airway tree caliber was 98 ± 11% predicted. Over a median of 5.0-yr follow-up interval, the change in FEV_1_ and FEV_1_/FVC was −40 ± 137 mL/yr and −0.009 ± 0.032 per year, respectively. Excluded SPIROMICS participants were more likely to be female and report fewer pack-years of smoking (Supplemental Table S1).

### Airway Tree Caliber Heterogeneity in the Absence of Standard COPD Risk Factors

The community-based nonsmoking participants without secondhand/occupational exposures or asthma included 289 MESA Lung participants (mean age 65 ± 8 yr, 58% female, mean FEV_1_/FVC 0.76 ± 0.09, mean airway tree caliber were 99 ± 9% predicted) and 204 CanCOLD participants (mean age 67 ± 11 yr, 41% female, mean FEV_1_/FVC 0.74 ± 0.08 mean airway tree caliber 96 ± 10% predicted) (Table 1).

**Figure 1.**
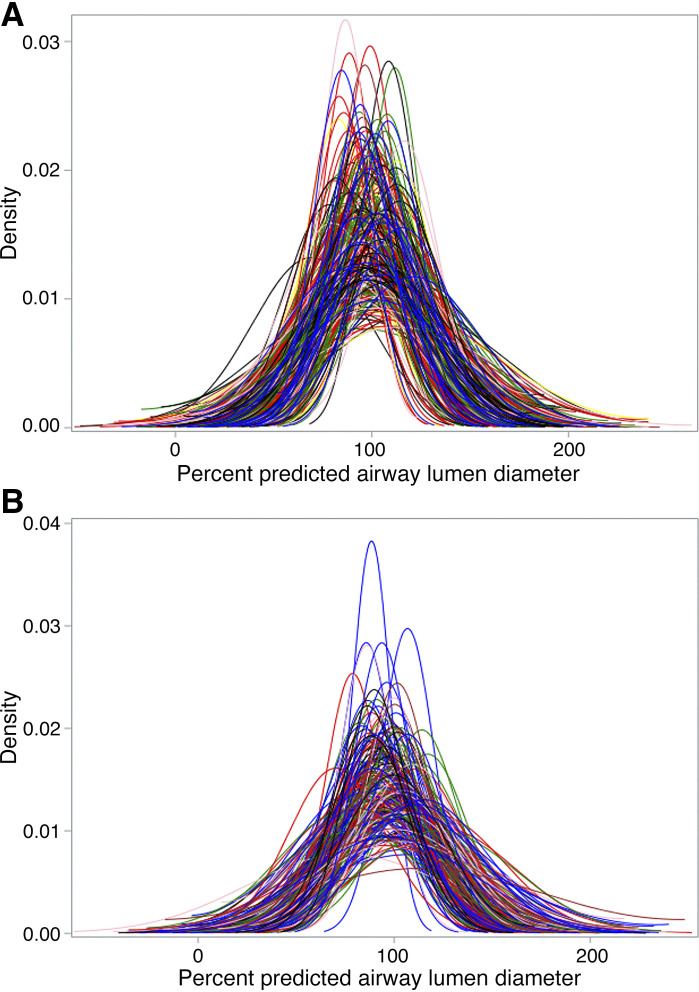
Airway tree caliber distributions among older adults free of standard COPD risk factors. Each colored curve depicts the distribution of percent-predicted airway lumen diameters within a nonsmoking participant free of secondhand smoke or occupational exposures or asthma diagnosis in MESA Lung (*A*) and CanCOLD (*B*). The SD metric quantifies the width of the curve, whereas the mean precent-predicted airway tree caliber quantifies the location of distribution along the *x*-axis. With increasing airflow obstruction and COPD prevalence, the mean percent-predicted airway tree caliber tends to be smaller (i.e., the central location of the Gaussian distribution tends to be less than 100%), whereas the SD of percent-predicted airway tree caliber tends to be larger (i.e., the width of Gaussian distribution tends to be wider). Airway lumen diameters were measured at 19 standard anatomical locations and percent-predicted values were calculated from externally validated airway-specific lumen diameter reference equations (9). Each participant’s airway tree caliber distribution is depicted as a kernel density estimate rather than a histogram to visualize the variations in distribution width. CanCOLD, Canadian Cohort of Obstructive Lung Disease; COPD, chronic obstructive pulmonary disease; MESA, Multi-Ethnic Study of Atherosclerosis.

**Figure 2.**
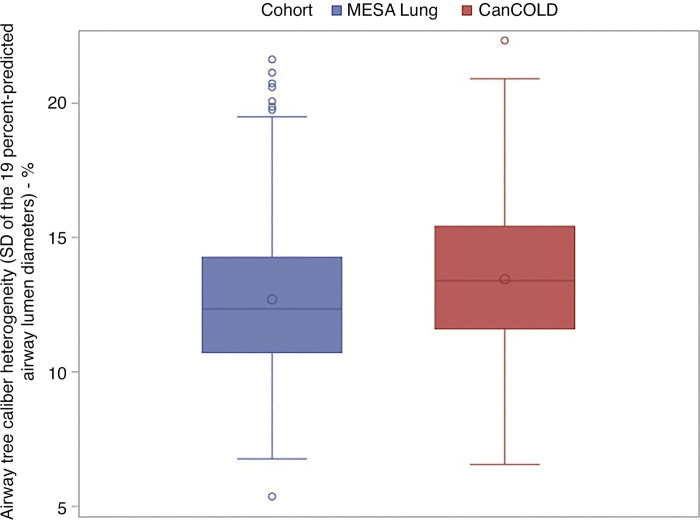
Box and whisker plot of airway tree heterogeneity among older adults free of standard COPD risk factors. Airway tree caliber heterogeneity was quantified as the standard deviation (SD) of percent-predicted airway lumen diameters. CanCOLD, Canadian Cohort of Obstructive Lung Disease; COPD, chronic obstructive pulmonary disease; MESA, Multi-Ethnic Study of Atherosclerosis.

**Figure 3.**
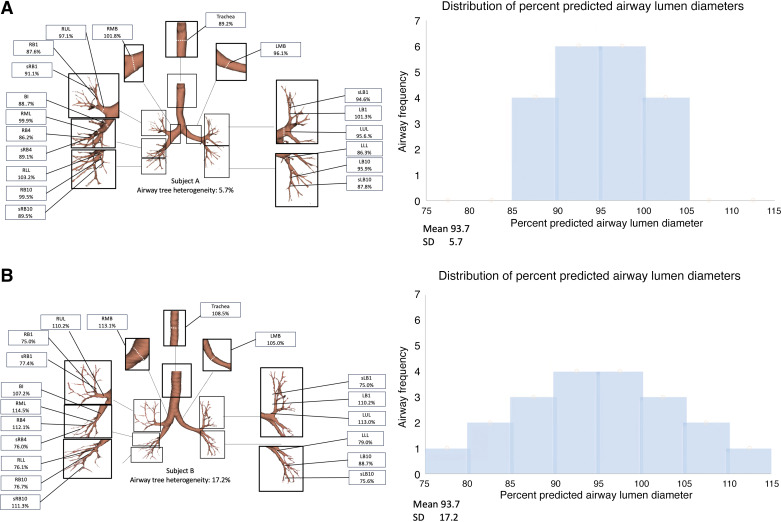
Depiction of airway tree lumen diameter heterogeneity assessment. Airway lumen diameters at 19 standard anatomical locations were measured from inspiratory chest CT images using Apollo Software. For each of the 19 standard anatomical airways, the percent-predicted airway caliber was calculated using externally validated sex-stratified airway-specific reference equations among nonsmoking adults free of clinical lung disease that included terms for total lung volume, age, and body height (*A*) (9). The distribution of percent-predicted airway lumen diameters was Gaussian (*B*) and could be summarized for each participant using the mean (an index of mean airway tree caliber) and standard deviation (an index of airway tree caliber heterogeneity). *Subject A* exhibits a mean airway tree caliber of 93.7% predicted with SD of 5.7%. *Subject B* also exhibits a mean airway tree caliber of 93.7% predicted but has an SD of 17.2%, representing comparatively higher airway tree caliber heterogeneity. See methods for additional details. CT, computed tomography.

The distribution of percent-predicted airway lumen diameters of each participant free of COPD risk factors and participants with COPD appeared Gaussian (Fig. 1 and Supplemental Fig. S2). Thus, airway tree caliber heterogeneity for each participant was quantified as the SD of their percent-predicted airway lumen diameters (Fig. 2). The Spearman correlation coefficient between airway tree caliber heterogeneity and mean airway tree caliber was 0.17 (95%CI: 0.05, 0.28; *P* = 0.004) in MESA Lung and 0.30 (95%CI: 0.16, 0.42; *P* < 0.0001) in CanCOLD. Figure 3 depicts representative airway trees from participants with low and high airway tree caliber heterogeneity.

### Airway Tree Caliber Heterogeneity, Baseline Airflow Obstruction, and COPD Status

**Figure 4.**
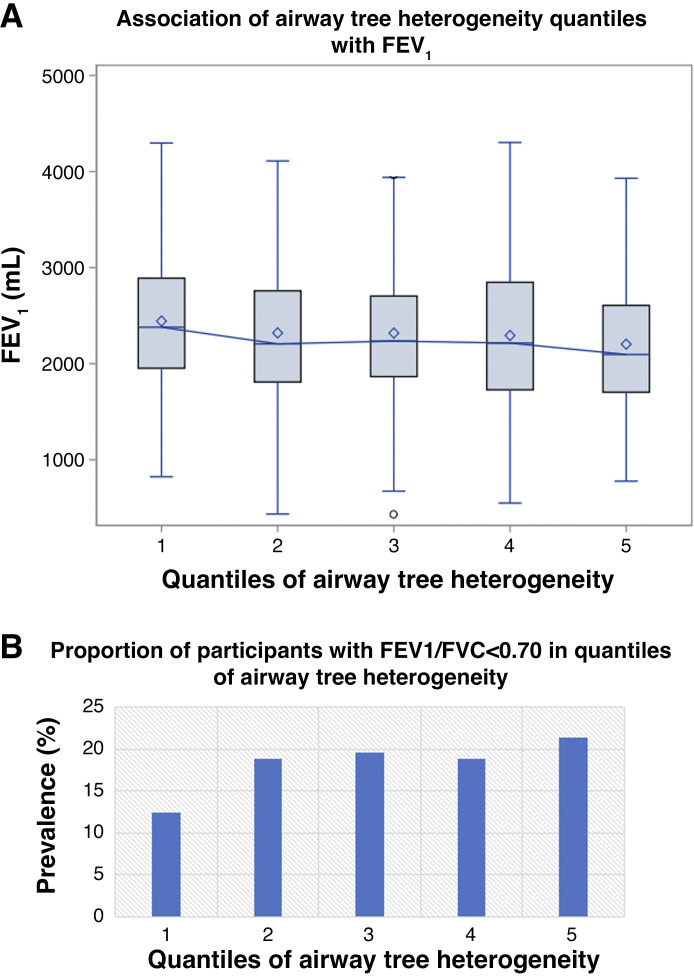
The association between FEV_1_, FEV_1_/FVC < 0.7 and airway tree heterogeneity. *A* and *B* show box and whisker plot and bar chart illustrating the association between FEV_1_, FEV_1_/FVC < 0.7, and airway tree heterogeneity, respectively. The diamonds and lower and upper bounds of the boxes denote the median, 25^th^ and 75^th^ percentiles, respectively; the whiskers define the upper and lower 25th percentiles and circle datapoints outside of these bounds. FEV_1_, forced expired volume in 1 second; FVC, forced vital capacity.

The association between FEV_1_, FEV_1_/FVC < 0.7, and airway tree heterogeneity are presented graphically in Fig. 4. Among all MESA Lung participants, those in the highest quartile of airway tree caliber heterogeneity exhibited lower FEV_1_ (adjusted mean difference: −125 mL, 95%CI: −171, −79; *P* < 0.0001), lower FEV_1_/FVC (adjusted mean difference: −0.01, 95%CI: −0.02, −0.01; *P* = 0.001), and higher odds of COPD (adjusted odds ratio: 1.42, 95%CI: 1.01, 2.02; *P* = 0.043) when compared with participants to the lowest quartile, independent of age, age^2^, sex, height, height^2^, race-ethnicity, mean airway tree caliber, tobacco exposures, occupational exposures, and asthma (Table 2).

**Table 2.** Airway tree caliber heterogeneity association with FEV_1,_ FEV_1_/FVC, and COPD

	Airway Tree Caliber Heterogeneity				
	Quartile 1	Quartile 2	Quartile 3	Quartile 4	Per 1-SD Increment
MESA (*n* = 2,505)					
FEV_1_, mL, means ± SD	2,421 ± 696	2,315 ± 704	2,311 ± 733	2,220 ± 725	
Mean difference (95% CI): Model 1	Reference	−54 (−99, −9) *P* = 0.020	−85 (−130, −39) *P* < 0.001	−149 (−196, −103) *P* < 0.0001	−64 (−80, −47) *P* < 0.0001
Mean difference (95% CI): Model 2	Reference	−53 (−97, −9) *P* = 0.018	−76 (−120, −32) *P* < 0.001	−125 (−171, −79) *P* < 0.0001	−56 (−72, −40) *P* < 0.0001
FEV_1_/FVC, means ± SD	0.75 ± 0.07	0.75 ± 0.08	0.74 ± 0.09	0.74 ± 0.09	
Mean difference (95% CI): Model 1	Reference	−0.00 (−0.01, 0.01) *P* = 0.410	−0.01 (−0.02, −0.00) *P* = 0.010	−0.02 (−0.03, −0.01) *P* < 0.0001	−0.01 (−0.01, −0.01) *P* < 0.0001
Mean difference (95% CI): Model 2	Reference	−0.00 (−0.01, 0.00) *P* = 0.376	−0.01 (−0.02, −0.00) *P* = 0.014	−0.01 (−0.02, −0.01) *P* = 0.001	−0.01 (−0.01, −0.00) *P* < 0.0001
FEV_1_/FVC<LLN, no. (%)	34 (5.4)	49 (7.8)	60 (9.6)	51 (8.1)	
Odds ratio (95% CI): Model 1	Reference	1.42 (0.88, 2.30) *P* = 0.631	1.95 (1.22, 3.10) *P* = 0.063	1.92 (1.18, 3.13) *P* = 0.095	1.31 (1.11, 1.54) *P* = 0.001
Odds ratio (95% CI): Model 2	Reference	1.39 (0.83, 2.31) *P* = 0.760	1.90 (1.16, 3.11) *P* = 0.062	1.70 (1.02, 2.84) *P* = 0.303	1.29 (1.09, 1.54) *P* = 0.004
FEV1/FVC < 0.7, no. (%)	92 (14.7)	118 (18.9)	135 (21.6)	126 (20.1)	
Odds ratio (95% CI): Model 1	Reference	1.32 (0.95, 1.83) *P* = 0.100	1.66 (1.20, 2.30) *P* = 0.002	1.71 (1.22, 2.39) *P* = 0.005	1.26 (1.12, 1.42) *P* < 0.0001
Odds ratio (95% CI): Model 2	Reference	1.19 (0.91, 1.81) *P* = 0.151	1.60 (1.14, 2.24) *P* = 0.006	1.42 (1.01, 2.02) *P* = 0.043	1.19 (1.05, 1.35) *P* = 0.006
CanCOLD (*n* = 1,297)					
FEV_1_, mL, means ± SD	2,604 ± 852	2,537 ± 848	2,466 ± 830	2,465 ± 853	
Mean difference (95% CI): Model 1	Reference	−58 (−151, 36) *P* = 0.226	−98 (−192, −3) *P* = 0.42	−223 (−321, −124) *P* < 0.0001	−102 (−136, −68) *P* < 0.0001
Mean difference (95% CI): Model 2	Reference	−62 (−151, 27) *P* = 0.172	−96 (−187, −6) *P* = 0.037	−206 (−301, −110) *P* < 0.0001	−91 (−124, −58) *P* < 0.0001
FEV_1_/FVC, means ± SD	0.72 ± 0.08	0.71 ± 0.08	0.72 ± 0.09	0.71 ± 0.09	
Mean difference (95% CI): Model 1	Reference	−0.01 (−0.02, 0.00) *P* = 0.185	−0.01 (−0.02, −0.00) *P* = 0.046	−0.02 (−0.03, −0.01) *P* < 0.001	−0.01 (−0.01, −0.00) *P* < 0.001
Mean difference (95% CI): Model 2	Reference	−0.01 (−0.02, 0.00) *P* = 0.159	−0.01 (−0.02, 0.00) *P* = 0.085	−0.02 (−0.03, −0.01) *P* = 0.003	−0.01 (−0.10, −0.00) *P* = 0.010
FEV_1_/FVC<LLN, no. (%)	69 (21.5)	99 (31.1)	97 (30.6)	109 (34.4)	
Odds ratio (95% CI): Model 1	Reference	1.88 (1.29, 2.74) *P* = 0.677	2.04 (1.40, 3.00) *P* = 0.254	2.70 (1.83, 3.98) *P* < 0.001	1.37 (1.20, 1.57) *P* < 0.0001
Odds ratio (95% CI): Model 2	Reference	1.98 (1.32, 2.96) *P* = 0.248	1.81 (1.20, 2.74) *P* = 0.663	2.43 (1.61, 3.68) *P* = 0.005	1.30 (1.13, 1.49) *P* < 0.001
FEV_1_/FVC < 0.7, no. (%)	134 (41.7)	166 (52.2)	156 (49.2)	162 (51.1)	
Odds ratio (95% CI): Model 1	Reference	1.72 (1.23, 2.39) *P* = 0.002	1.82 (1.30, 2.55) *P* < 0.001	1.95 (1.38, 2.76) *P* < 0.001	1.27 (1.11, 1.44) *P* < 0.001
Odds ratio (95% CI): Model 2	Reference	1.72 (1.21, 2.44) *P* = 0.003	1.61 (1.12, 2.31) *P* = 0.010	1.65 (1.14, 2.38) *P* = 0.003	1.17 (1.03, 1.34) *P* = 0.018
SPIROMICS (*n* = 2,730)					
FEV_1_, mL, means ± SD	1,950 ± 855	1,950 ± 867	1,841 ± 923	1,861 ± 923	
Mean difference (95% CI): Model 1	Reference	−51 (−117, 15) *P* = 0.131	−163 (−229, −97) *P* < 0.0001	−253 (−320, −187) *P* < 0.0001	−112 (−136, −88) *P* < 0.0001
Mean difference (95% CI): Model 2	Reference	−42 (−107, 22) *P* = 0.201	−150 (−214, −85) *P* < 0.0001	−238 (−303, −173) *P* < 0.0001	−106 (−130, −83) *P* < 0.0001
FEV_1_/FVC, means ± SD	0.69 ± 0.15	0.58 ± 0.16	0.57 ± 0.17	0.56 ± 0.17	
Mean difference (95% CI): Model 1	Reference	−0.02 (−0.03, −0.01) *P* = 0.003	−0.04 (−0.05, −0.03) *P* < 0.0001	−0.06 (−0.07, −0.04) *P* < 0.0001	−0.02 (−0.03, −0.02) *P* < 0.0001
Mean difference (95% CI): Model 2	Reference	−0.02 (−0.03, −0.01) *P* = 0.006	−0.04 (−0.05, −0.02) *P* < 0.0001	−0.05 (−0.07, −0.04) *P* < 0.0001	−0.02 (−0.03, −0.02) *P* < 0.0001
FEV_1_/FVC< LLN, no. (%)	358 (52.9)	371 (54.8)	403 (60.1)	406 (60.1)	
Odds ratio (95% CI): Model 1	Reference	1.23 (0.96, 1.57) *P* = 0.022	1.72(1.33, 2.22) *P* = 0.050	2.20 (1.69, 2.85) *P* < 0.0001	1.39 (1.26, 1.53) *P* < 0.0001
Odds ratio (95% CI): Model 2	Reference	1.22 (0.95, 1.57) *P* = 0.025	1.71 (1.32, 2.22) *P* = 0.056	2.18 (1.67, 2.85) *P* < 0.0001	1.38 (1.26, 1.53) *P* < 0.0001
FEV_1_/FVC < 0.7	429 (63.5)	453 (66.9)	460 (68.6)	457 (67.6)	
Odds ratio (95% CI): Model 1	Reference	1.42 (1.09, 1.84) *P* = 0.003	1.64 (1.26, 2.14) *P* < 0.001	2.11 (1.60, 2.78) *P* < 0.0001	1.34 (1.21, 1.48) *P* < 0.0001
Odds ratio (95% CI): Model 2	Reference	1.41 (1.08, 1.83) *P* = 0.011	1.64 (1.25, 2.15) *P* < 0.001	2.07 (1.57, 2.74) *P* < 0.0001	1.32 (1.19, 1.47) *P* < 0.0001

The mean differences and odds ratios were estimated by fitting linear and logistic regression models, respectively.

Model 1 covariables: age, age^2^, sex, height, height^2^, race-ethnicity, and mean airway tree caliber.

Model 2 covariables: model 1 variables + cigarette smoking status, pack-yr, pipe smoking status, pipe-years, cigar smoking status, cigar-years, secondhand smoke exposure, and occupational exposure to vapor-gas, dust, or fumes, and asthma diagnosis. Airway tree caliber heterogeneity was quantified as the standard deviation (SD) of percent-predicted airway diameters measured at 19 standard anatomical locations.

CanCOLD, Canadian cohort of obstructive lung disease; CI, confidence interval; COPD, chronic obstructive pulmonary disease; FEV_1_, forced expired volume in 1 second; FVC, forced vital capacity; LLN, lower limit of normal; MESA, Multi-Ethnic study of atherosclerosis; SPIROMICS, subpopulations and intermediate outcome measures in COPD study.

Among all CanCOLD participants, the highest quartile of airway tree caliber heterogeneity was also associated with lower FEV_1_ (adjusted mean difference: −206 mL, 95%CI: −301, −110; *P* < 0.0001), lower FEV_1_/FVC (adjusted mean difference: −0.02, 95%CI: −0.03, −0.01; *P* = 0.003), and higher odds of COPD (adjusted odds ratio: 1.65; 95%CI: 1.14, 2.38; *P* = 0.003) when compared with the lowest quartile in the main adjusted model (Table 2).

Among SPIROMICS participants with 20+ pack-yr of smoking, the highest quartile of airway tree caliber heterogeneity was associated with lower FEV_1_ (adjusted mean difference: −238 mL, 95%CI: −303, −173; *P* < 0.0001), lower FEV_1_/FVC (adjusted mean difference: −0.05, 95%CI: −0.07, −0.04; *P* < 0.0001), and higher COPD odds (adjusted odds ratio: 2.07, 95%CI: 1.57, 2.74; *P* < 0.0001) when compared with participants in the lowest quartile in the main adjusted model (Table 2).

### Airway Tree Caliber Heterogeneity and Longitudinal Change in Spirometry

Among MESA Lung participants, there was no evidence that airway tree caliber heterogeneity was associated with longitudinal change in spirometry. Comparing participants in the highest quartile of airway tree caliber heterogeneity to those in the lowest quartile, there was no difference in the annualized change in FEV_1_ (adjusted mean difference: 10 mL/yr 95%CI: −34, 54; *P* = 0.717) or FEV_1_/FVC (adjusted mean difference: 0.007; 95%CI: −0.004, 0.017; *P* = 0.204). Consistent findings were observed in CanCOLD and SPIROMICS (Table 3).

**Table 3.** Airway tree caliber heterogeneity associations with longitudinal lung function

	Airway Tree Caliber Heterogeneity				
	Quartile 1	Quartile 2	Quartile 3	Quartile 4	Per 1-SD Increment
MESA (*n* = 2,505)					
Annualized FEV_1_ change in mL, means ± SD	−34 ± 28	−32 ± 30	−34 ± 33	−33 ± 33	
Mean difference (95%CI): Model 1	Reference	−58 (−101, −14) *P* = 0.009	−48 (−93, −4) *P* = 0.034	8 (−36, 52) *P* = 0.717	0.2 (−16, 16) *P* = 0.978
Mean difference (95%CI): Model 2	Reference	−56 (−100, −13) *P* = 0.011	−45 (−90, −1) *P* = 0.048	10 (−34, 54) *P* = 0.717	1 (−14, 17) *P* = 0.872
Annualized FEV_1_/FVC change, means ± SD	−0.00 ± 0.01	−0.00 ± 0.01	−0.00 ± 0.01	−0.00 ± 0.01	
Mean difference (95%CI): Model 1	Reference	0.01 (−0.01, 0.02) *P* = 0.367	0.00 (−0.01, 0.01) *P* = 0.859	0.01 (−0.00, 0.02) *P* = 0.164	−0.00 (−0.00, 0.01) *P* = 0.309
Mean difference (95%CI): Model 2	Reference	0.00 (−0.01, 0.01) *P* = 0.446	0.00 (−0.01, 0.01) *P* = 0.908	0.01 (−0.00, 0.02) *P* = 0.204	0.00 (−0.00, 0.01) *P* = 0.327
CanCOLD (*n* = 1,045)					
Annualized FEV_1_ change in mL, means ± SD	−32 ± 78	−38 ± 76	−34 ± 84	−40 ± 77	
Mean difference (95%CI): Model 1	Reference	6 (−18, 30) *P* = 0.645	−17 (−41, 7) *P* = 0.158	7 (−18, 32) *P* = 0.568	−2 (−11, 7) *P* = 0.721
Mean difference (95%CI): Model 2	Reference	3 (−21, 27) *P* = 0.802	−19 (−43, 5) *P* = 0.127	5 (−20, 30) *P* = 0.691	−2 (−11, 7) *P* = 0.668
Annualized FEV_1_/FVC change, means ± SD	−0.00 ± 0.02	−0.00 ± 0.02	−0.00 ± 0.02	−0.00 ± 0.02	
Mean difference (95%CI): Model 1	Reference	−0.00 (−0.01, 0.00) *P* = 0.904	−0.00 (−0.01, 0.00) *P* = 0.595	0.01 (−0.00, 0.01) *P* = 0.619	0.00 (−0.00, 0.00) *P* = 0.169
Mean difference (95%CI): Model 2	Reference	−0.00 (−0.01, 0.00) *P* = 0.678	−0.00 (−0.01, 0.00) *P* = 0.483	0.00 (−0.00, 0.01) *P* = 0.116	0.00 (−0.00, 0.00) *P* = 0.254
SPIROMICS (*n* = 2,144)					
Annualized FEV_1_ change in mL, means ± SD	−50 ± 124	−28 ± 147	−41 ± 125	−43 ± 143	
Mean difference (95%CI): Model 1	Reference	−0.4 (−7, 6) *P* = 0.894	1 (−6, 8) *P* = 0.759	−1 (−8, 6) *P* = 0.699	0.4 (−2, 3) *P* = 0.747
Mean difference (95%CI): Model 2	Reference	0.4 (−6, 7) *P* = 0.892	2 (−5, 8) *P* = 0.656	−0.1 (−7, 7) *P* = 0.968	1 (−1, 3) *P* = 0.433
Annualized FEV_1_/FVC change, means ± SD	−0.01 ± 0.03	−0.01 ± 0.03	−0.01 ± 0.03	−0.01 ± 0.03	
Mean difference (95%CI): Model 1	Reference	−0.00 (−0.00, 0.00)*P* = 0.347	0.00 (−0.00, 0.00) *P* = 0.477	−0.00 (−0.00, 0.00) *P* = 0.425	−0.00 (−0.00, 0.00) *P* = 0.140
Mean difference (95%CI): Model 2	Reference	−0.00 (−0.00, 0.00) *P* = 0.371	0.00 (−0.00, 0.00) *P* = 0.620	−0.00 (−0.00, 0.00) *P* = 0.614	−0.00 (−0.00, 0.00) *P* = 0.285

The mean differences were estimated by fitting linear regression models.

Model 1 covariables: age, age^2^, sex, height, height^2^, race-ethnicity, and mean airway tree caliber.

Model 2 covariables: model 1 variables + cigarette smoking status, pack-yr, pipe smoking status, pipe-years, cigar smoking status, cigar-years, secondhand smoke exposure, and occupational exposure to vapor-gas, dust, or fumes, and asthma diagnosis. Airway tree caliber heterogeneity was quantified as the standard deviation (SD) of percent-predicted airway diameters measured at 19 standard anatomical locations.

CanCOLD, Canadian cohort of obstructive lung disease; CI, confidence interval; FEV_1_, forced expired volume in 1 second; FVC, forced vital capacity; MESA, Multi-Ethnic study of atherosclerosis; SPIROMICS, subpopulations and intermediate outcome measures in COPD study.

### Sensitivity Analyses

Restricted cubic splines did not improve model fit and associations were consistent when airway tree caliber heterogeneity was modeled per 1-SD increment (i.e., linear) and across quartiles (Tables 2 and 3). Observations were consistent in unweighted CanCOLD and unimputed MESA samples (Supplemental Table S2). Analyses stratified by ever and never smoking strata in MESA Lung and CanCOLD were consistent, except the association between airway tree caliber heterogeneity and COPD was attenuated among nonsmokers (Supplemental Table S3 and S4). There was no statistical evidence of modification of airway tree caliber heterogeneity associations by smoking status (*P*-interaction > 0.48) or mean airway tree caliber (*P*-interaction > 0.11). The coefficient of variation of percent-predicted airway lumen diameters, which combines airway tree heterogeneity and mean caliber into a single ratio measure, yielded consistent results, with even larger magnitude estimates of association with airflow obstruction and COPD (Supplemental Table S5). Race-ethnicity-stratified analyses in MESA Lung and analyses additionally adjusted for study sites in all cohorts were consistent (Supplemental Table S6–S7).

Defining COPD by spirometry and respiratory symptoms yielded similar associations (Supplemental Table S8).

## DISCUSSION

Among older adults in two community-based samples and a case-control study of heavy smokers, conducting airway tree caliber heterogeneity quantified by CT was associated with baseline airflow obstruction and COPD independent of age, sex, height, race-ethnicity, and mean airway tree caliber, but was not associated with longitudinal lung function decline. These observations suggest that conducting airway tree caliber heterogeneity is a structural trait associated with low baseline lung function and normal decline trajectory that may contribute to prevalent COPD among older adults.

Initially inferred from maximum expiratory airflow variation among healthy adults, Green and colleagues (46) hypothesized that variation in airway tree caliber arising early in life was an important host susceptibility factor for COPD (“dysanapsis”). In support of this hypothesis, variation in mean airway tree caliber and its association with airflow obstruction is manifested by early adulthood and among older adults is independently associated with COPD prevalence but normal lung function decline (9, 39, 47). The present study adds to the emerging clinical relevance of native airway tree structure by showing that airway tree caliber heterogeneity is associated with airflow obstruction and COPD independent of mean airway tree caliber and other standard COPD risk factors. Whether airway tree caliber heterogeneity and mean airway tree caliber arise from the same underlying developmental mechanism postulated by Green and colleagues is uncertain, though we note that the correlation between these two structural traits was relatively weak.

The mechanisms by which airway tree caliber heterogeneity relates to airflow obstruction and COPD were not assessed in this study. Several inert gas washout studies have suggested that ventilation distribution inhomogeneity is a feature of chronic obstructive lung diseases and, leveraging fundamental laws of gas flow and mixing (48, 49), these studies have articulated three principal mechanisms that include convection-dependent inhomogeneity arising from the conducting airways, diffusion-limitation inhomogeneity arising at the distal acinar level, and diffusion convection-interaction-dependent inhomogeneity arising at the diffusion-convection front, which is estimated to occur at the acinar entrance (17, 18, 20–22, 26). The present study complements these important works by *1*) providing a new method for quantifying conducting airway tree caliber heterogeneity in vivo and *2*) applying this method to quantify and replicate the presence of conducting airway tree caliber heterogeneity and its association with airflow obstruction in large and diverse samples. Combined with the in vivo quantification of mean conducting airway tree caliber, one can probe (i.e., partition) the independent contributions of both structural properties of the airway tree to obstructive pathophysiology. Application of these methods to cohorts with inert gas washout measurements may validate certain postulated mechanisms of ventilation inhomogeneity and, potentially, find some clinical utility in endo-phenotyping the seemingly heterogeneous cluster of chronic obstructive lung diseases.

Imaging studies have demonstrated heterogeneity of inhaled gas distribution in obstructive lung diseases and related these deficits to regional airway tree caliber narrowing in small-to-moderate samples (27–31, 50–52). The present study complements these works by demonstrating conducting airway tree caliber heterogeneity and its association with airflow obstruction across the spectrum of obstructive lung disease severity and, critically, even among individuals free of traditional obstructive lung disease risk factors (e.g., tobacco smoke exposures, asthma). These observations suggest developmental processes may contribute to airway tree caliber heterogeneity-associated airflow obstruction (51).

Airway tree caliber heterogeneity in the present study was not associated with lung function decline. Similar to prior observations on mean airway tree caliber, this finding suggests that airway tree caliber heterogeneity is another structural trait associated with the “low-baseline-normal-decline” lung function trajectory experienced by 50% of older adults with COPD (9, 14, 53). Indeed, the percent-predicted airway caliber coefficient of variation, which combines heterogeneity and mean airway tree caliber into a single quantitative measure exhibited the largest association estimates with baseline airflow obstruction and COPD prevalence. Together, these observations suggest that the dysanaptic pathway to COPD may not be limited to developmental differences in mean airway tree caliber, but rather may also include differences in airway tree caliber heterogeneity.

The etiology of airway tree caliber heterogeneity was not assessed in this study. We note, however, that variation in mean airway tree caliber has been demonstrated among young nonsmoking adults free of lung disease (39), suggesting that this structural trait arises earlier in life. We also note that the airway caliber reference equations did not include terms for race-ethnicity to avoid normalization of potential sources of disparities related to race-ethnicity (54), nevertheless associations were consistent within race-ethnic strata. These observations suggest that airway tree caliber associations with airflow obstruction and COPD are not related to race-ethnic differences. Investigating the etiology of airway tree caliber earlier in life will likely require radiation-free (or lower dose radiation) methods to quantify lung structure heterogeneity in children.

The findings from this study must be considered in the context of its limitations. First, unmeasured, imprecisely measured, or differential susceptibility to standard COPD risk factors associated with airway tree caliber heterogeneity may inflate association estimates. We think this is unlikely since there were detailed and standardized COPD risk factor assessments and analyses restricted to people who never smoked yielded similar results. Second, airway tree assessment was limited to 19 central airways. This approach minimized airway detection bias related to CT resolution or anatomical variation in airway tree branch patterns (8). Third, airway caliber inhomogeneity and airflow obstruction may be two aspects of obstructive lung disease that are not linked mechanistically. Establishing a causal mechanistic link between airway inhomogeneity and lung function decline requires experimental interruption of airway inhomogeneity while observing lung function over time and interruption of lung function decline while observing airway inhomogeneity. Fourth, the lack of association with lung function decline may be related to power (55). Fifth, inferences about the quality of inspiration at CT based upon a comparison between CT total lung volume (CT-TLV) and spirometry (e.g., FVC) are limited. Future studies assessing modifiable etiological factors, structure-clinical outcome relationships, and regional/peripheral airway tree heterogeneity are warranted (56).

### Conclusion

Among community-dwelling older adults and heavy smokers with and without COPD, airway tree caliber heterogeneity was associated with baseline airflow obstruction and COPD independent of mean airway tree caliber but was not associated with prospective change in lung function. These findings suggest that native airway tree caliber heterogeneity may be a host structural factor relevant to COPD.

## DATA AVAILABILITY

Data will be made available upon reasonable request.

## GRANTS

This study was supported by NIH/National Heart, Lung, and Blood Institute: R01-HL130506 (to B. M. Smith), R01-HL077612 (to R. G. Barr), R01-HL093081 (to R. G. Barr), CIHR: PJT-162335 (to B. M. Smith), K23ES030725 (to C. Sack), and Vanier Canada Graduate Scholarship (to M. Vameghestahbanati). MESA was supported by contracts 75N92020D00001, HHSN268201500003I, N01-HC-95159, 75N92020D00005, N01-HC-95160, 75N92020D00002, N01-HC-95161, R01-HL077612, R01-HL093081,75N92020D00003, N01-HC-95162, 75N92020D00006, N01-HC-95163, 75N92020D00004, N01-HC-95164, 75N92020D00007, N01-HC-95165, N01-HC-95166, N01-HC-95167, N01-HC-95168, and N01-HC-95169 from the National Heart, Lung, and Blood Institute, and by grants UL1-TR-000040, UL1-TR-001079, and UL1-TR-001420 from the National Center for Advancing Translational Sciences (NCATS). MESA Air was developed under the Science to Achieve Results (STAR) research assistance Agreement Nos. RD831697 (MESA Air) and RD-83830001 (MESA Air Next Stage), awarded by the US Environmental Protection Agency (EPA). CanCOLD was supported by the Canadian Respiratory Research Network; industry partners: Astra Zeneca Canada Ltd; Boehringer Ingelheim Canada Ltd; GlaxoSmithKline Canada Ltd; and Novartis. Previous funding partners are the CIHR (CIHR/Rx&D Collaborative Research Program Operating Grants 93326); the Respiratory Health Network of the Fonds de la recherche en santé du Québec (FRSQ); industry partners: Almirall; Merck Nycomed; Pfizer Canada Ltd; and Theratechnologies. SPIROMICS was supported by contracts from the NIH/NHLBI (HHSN268200900013C, HHSN268200900014C, HHSN268200900015C, HHSN268200900016C, HHSN268200900017C, HHSN268200900018C, HHSN268200900019C, and HHSN268200900020C), grants from the NIH/NHLBI (U01 HL137880, U24 HL141762, and R01-HL093081), and supplemented by contributions made through the Foundation for the NIH and the COPD Foundation from AstraZeneca/MedImmune; Bayer; Bellerophon Therapeutics; BoehringerIngelheim Pharmaceuticals, Inc.; Chiesi Farmaceutici S.p.A.; Forest Research Institute, Inc.; GlaxoSmithKline; Grifols Therapeutics, Inc.; Ikaria, Inc.; Novartis Pharmaceuticals Corporation; Nycomed GmbH; ProterixBio; Regeneron Pharmaceuticals, Inc.; Sanofi; Sunovion; Takeda Pharmaceutical Company; and Theravance Biopharma and Mylan.

## DISCLAIMERS

The views expressed in this document have not been formally reviewed by the EPA and are solely those of the authors. The EPA does not endorse any products or commercial services mentioned in this publication.

## DISCLOSURES

No conflicts of interest, financial or otherwise, are declared by the authors.

## AUTHOR CONTRIBUTIONS

M.V. and B.M.S. conceived and designed research; M.V. analyzed data; M.V. and B.M.S. interpreted results of experiments; M.V. prepared figures; M.V. drafted manuscript; M.V. and B.M.S. edited and revised manuscript; M.V., L.K., E.A.H., M.K., N.B.A., E.A., A.B., Q.H., J.C.H., D.R.J., A.L., F.M., E.D.M., C.S., D.S., K.E.W., A.W., D.C., C.C., M.H., P.W., W.C.T., J.B., R.G.B., and B.M.S., approved final version of manuscript.
